# Natural and human-made disaster and associated health outcomes among community-dwelling older adults in India: Findings from LASI, 2017–18

**DOI:** 10.1371/journal.pone.0307371

**Published:** 2024-07-18

**Authors:** T. Muhammad, Manacy Pai, Chanda Maurya, Shobhit Srivastava, Manish Kumar

**Affiliations:** 1 Center for Healthy Aging, The Pennsylvania State University, University Park, Pennsylvania, United States of America; 2 Department of Sociology and Criminology, Kent State University, Kent, Ohio, United States of America; 3 Department of Survey Research and Data Analytics, International Institute for Population Sciences, Mumbai, India; Dayeh University, TAIWAN

## Abstract

**Background:**

Droughts, flash floods, rail accidents, and riots are relatively regular occurrences for those living in many low- and middle-income countries like India. While such natural and human-made disasters put everyone in harm’s way, their toll on specific segments of society–like older adults–is the heaviest. Therefore, in this study, we examine (1) the prevalence of natural and human-made disasters in India and (2) the association between natural and human-made disasters and several physical and mental health outcomes among older Indians.

**Methods:**

A cross-sectional study was conducted utilizing data come from the 2017–18 wave 1 of the nationally representative Longitudinal Ageing Study in India, comprising a sample of 29,333 older adults (14,120 males and 15,213 females) aged 60 years and above. Multivariate random intercept multilevel logistic regression analysis is used to examine the association between natural and human-made disasters and poor self-rated health, difficulty in activities of daily living, difficulty in instrumental activities of daily living, communicable diseases, non-communicable diseases, depressive symptoms, and psychiatric disorder.

**Results:**

Overall, 3.58% of older adults reported that they have encountered any type of natural or human-made disaster in the past five years. Compared to those who did not experience any (natural or human-made) disaster, older adults who experienced any disaster had a higher prevalence of poor self-rated health (33.4% vs 23.31%), difficulty in activities of daily living (33.94% vs 23.00%), difficulty in instrumental activities of daily living (60.09% vs 47.70%), communicable diseases (49.57% vs 25.86%), depressive symptoms (17.30% vs 8.06%) and psychiatric disorders (3.42% vs 2.78%). After adjusting for the selected variables and the contextual effect, the odds of poor self-rated health (1.64 [1.40, 1.92]), difficulty in activities of daily living and instrumental activities of daily living (1.89 [1.61, 2.21] and 1.63 [1.40, 1.89]), communicable and non-communicable diseases (2.12 [1.83, 2.46] and 1.38 [1.20, 1.60]), depressive symptoms and psychiatric disorder (1.67 [1.55, 2.05] and 1.52 [1.33, 2.18]) were significantly higher among older adults who experienced a natural or human-made disaster than their counterparts without such an experience.

**Conclusions:**

Relative to their non-exposed counterparts, older Indians who survived natural or human-made disasters endured an inflated risk of poor self-rated health, functional difficulties, communicable and non-communicable diseases, depressive symptoms, and psychiatric disorders. As such, post-disaster efforts should be grounded in policies and programs that address disaster-related trauma and diseases and improve the functional, physical, and psychological facets of health among older disaster survivors.

## Background

The World Health Organization defines a *disaster* as any event that disrupts the daily functioning of a community or society, causing material, economic, or environmental losses, and overwhelming local capacity [1]. Disasters can be natural (e.g., earthquakes, tornadoes, and hurricanes) and human-made (e.g., armed conflicts, nuclear accidents, industrial explosions, infrastructural failures, environmental pollution) [1]. According to a National Portal of India report, 27 of 36 state and union territories remain prone to natural disasters [2]. Almost 60% of the Indian subcontinent remains vulnerable to flooding and earthquakes, 68% of the usable land is at risk of drought [3], and about 8% faces the risk of cyclones [4, 5]. In addition to natural calamities and the recent virus pandemic of COVID-19, Indians have lost lives and livelihoods to rail accidents, bridge collapses, public unrest, riots, and terrorism [4]. While such natural and human-made disasters put everyone in harm’s way, their toll on specific segments of society, like older adults, is the heaviest [6, 7].

### Conceptual framework

We use the fundamental cause theory (FCT) [8] to argue that older adults are particularly susceptible to disasters and their resulting consequences. FCT theorists Link and Phelan [8] argue that social factors, such as socioeconomic status (SES), are associated with a multitude of resources that can be used to prevent risks, implement preventive measures, and maintain and enhance health, making them fundamental and persistent causes of disease. Higher-SES persons have access to “flexible” or “tranferrable” resources, like knowledge, power, high social stature, and ties to mainstream society, that they can use to their advantage in times of need to protect health [8]. Here, we argue that, like SES, age is a social factor that shapes access to essential resources needed to protect oneself from disasters. Like low SES, old age, which is a stigmatized social status [9], increases exposure to multiple risk factors (e.g., chronic disease, physical frailty, cognitive decline), and increasing age often is attached to loss of vital resources, like power, prestige, and mainstream connections. Such losses, coupled with sensory decline, driving limitations [10], and decreasing social networks [11, 12] undermine both older adults’ ability to prepare for disasters and cope with post-disaster consequences. Specifically, FCT posits that social disparities play a crucial role in shaping both the causes and distribution of health outcomes, and underscores the importance of ensuring equal distribution of resources to mitigate prevailing health risks and render fair access to health advantages across all communities [13, 14].

### Consequences of disasters for older adults

When disasters devastate homes, prevent access to social services, and mandate evacuations or relocations, older individuals are rendered particularly vulnerable due to the interruptions such conditions bring to their health care [15]. A recent study found that the cyclones, floods, droughts, and heatwaves in Odisha, India, most affected the vulnerable segments of the population, including people with disabilities and older adults [16]. Comparably, studies conducted following the 2015 Chennai floods found that several older patients living in the affected areas experienced insufficient access to medications for cardiometabolic conditions [17]. Older people in West Bengal’s rural areas and the slums of Kolkata endured extreme temperatures leading to heat exhaustion [18]. Disaster exposure affects older adults’ physical and functional health in myriad ways, including weight gain, fatigue, frailty, and deficits in physical and social activity [19].

Human-made disasters also take a toll on older adults. During political, social, or health crises, older persons experience negative emotions due to social marginalization. COVID-19, for instance, increased instances of ageism. Such instances ranged from disparaging slogans like “Boomer remover” and “stay home” to the well-intentioned but demeaning messages to sequester indefinitely to the more obvious cases of discrimination like age-based rationing of life-saving treatments [20–25]. Such attitudes are often internalized [26], discourage social interactions and activity [27, 28], and may ultimately damage health. As such, compared to their younger peers, older adults have a higher prevalence of anxiety, sadness, and posttraumatic stress disorder (PTSD) in response to human-generated calamities such as pandemics, protests, riots, armed conflicts, and acts of hate and terrorism [29, 30].

**Fig 1:**
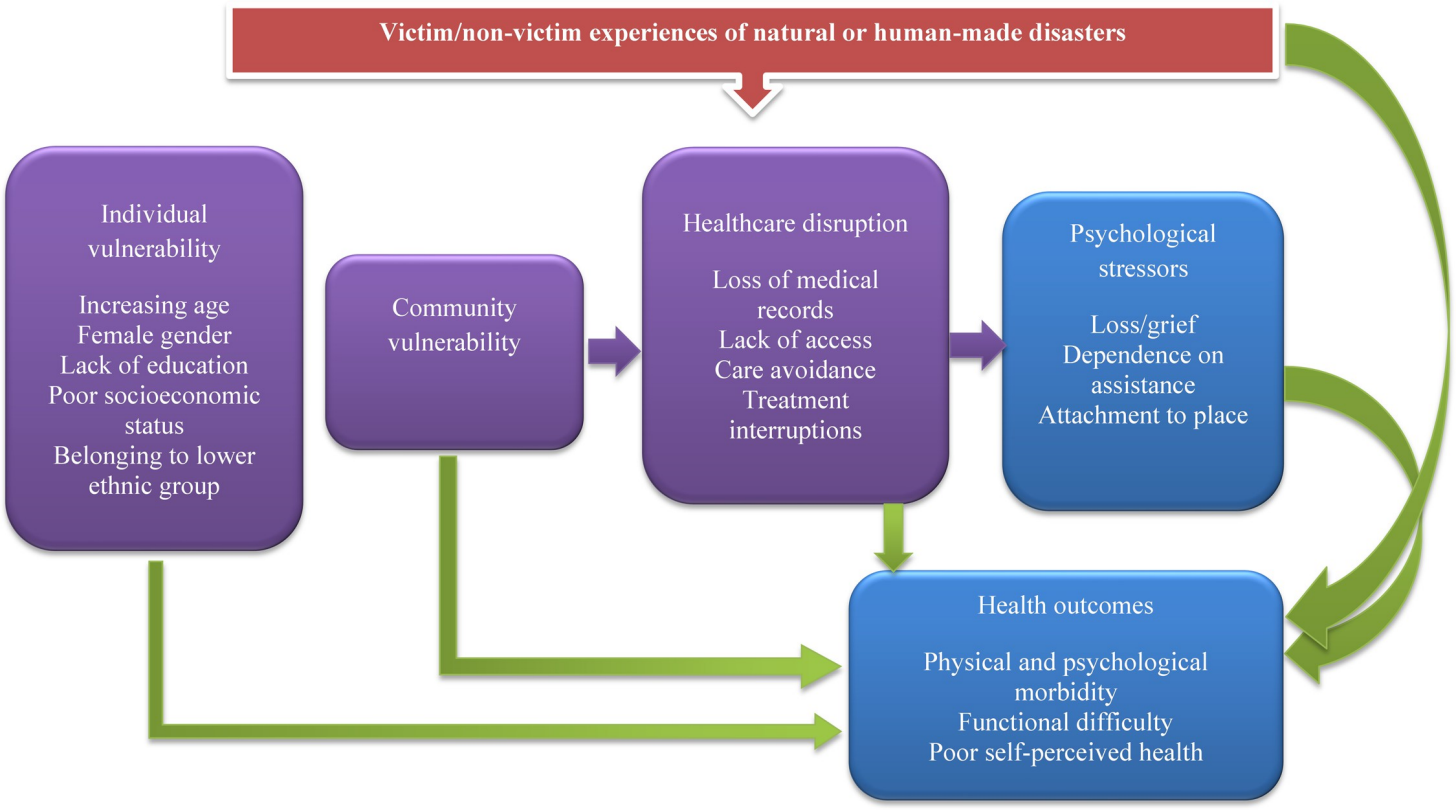
Conceptual framework of the study.

Existing studies find adverse health repercussions of natural and human-made disasters among older adults [31, 32]. However, such studies are scarce in LMICs like India where the proportion of older adults is rapidly increasing. As part of preparing all the states and union territories of the country in implementing the National Disaster Management Act, 2005, a comprehensive national plan was developed in India in 2016 [33]. The plan provides a holistic guidance for disaster risk reduction, mitigation, preparedness, response, recovery and rehabilitation, and suggests an optimum preparedness strategy for vulnerable populations including older adults who may be faced with multiple age-related limitations. Paying heed to this, we examine (1) the statewide prevalence of natural and human-made disasters in India and (2) the association between such disasters and poor self-rated health (SRH), difficulty in activities of daily living (ADL) and instrumental activities of daily living (IADL), communicable diseases (CDs), and non-communicable diseases (NCDs), depressive symptoms and psychiatric disorders among older Indians. We expect a positive association between natural or human-made disasters and each of the study outcomes. Fig 1 provides the conceptual framework for our study, grounded in FCT, presenting the direct effects of disasters on adverse health outcomes as and their indirect effects via increased individual, household, and community level vulnerabilities that lead to various psychological stressors which ultimately result in more adverse health outcomes.

## Methods

### Study design and participants

A cross-sectional study design was adopted. The study utilized publicly available secondary data from the Longitudinal Ageing Study in India (LASI- wave 1) conducted during 2017–18, which investigates the health, economic, and social determinants and consequences of population aging in India [34]. The sample in the LASI survey included 72,250 individuals aged 45 and above and their spouses regardless of age across all states and union territories of India except Sikkim. Households with at least one member aged 45 and above were taken as the eventual unit of observation. The data provides scientific evidence on demographics, household economic status, chronic health conditions, symptoms-based health conditions, functional and mental health, biomarkers, health care utilization, work and employment. It enables cross-state analyses and cross-national analyses of aging, health, economic status, and social behavior, and has been designed to evaluate the effect of changing policies and behavioral outcomes in India. Detailed information on the sampling frame is available in the LASI wave-1 Report [34]. After dropping the missing cases on the key variable, the final sample comprised 29,333 older adults (14,120 males and 15,213 females) aged 60 years and above.

During the LASI survey, written informed consent was obtained from all participants and their informants. The Indian Council of Medical Research (ICMR) extended the necessary guidelines and ethics approval for undertaking the LASI survey. All methods were carried out in accordance with the relevant guidelines and regulations of the ICMR.

#### Outcome variables

There were seven health outcome variables and all of them were dichotomized in the study. SRH was coded as good which includes very good, good and fair and, poor which includes poor and very poor. SRH is a subjective measure of one’s judgments about the personal health status, is considered the valuable indicator of long-term health status, and predicts illness and disability in the future [35]. ADL is a term used to refer to normal daily self-care activities (such as movement in bed, changing position from sitting to standing, feeding, bathing, dressing, grooming, personal hygiene etc.) The ability or inability to perform ADLs is used to measure a person’s functional status, especially in the case of people with disabilities and the older adults; and difficulty in ADL was coded as yes representing the presence of at least one difficulty. IADL are not necessarily related to the fundamental functioning of a person, but they let an individual live independently in a community. Respondents were asked if they were having any difficulties that were expected to last more than three months, such as preparing a hot meal, shopping for groceries, making a telephone call, taking medications, doing work around the house or garden, managing money (such as paying bills and keeping track of expenses), and getting around or finding an address in unfamiliar places; and difficulty in IADL was coded as yes representing the presence of at least one difficulty.

CDs among older adults were calculated using ten major communicable diseases. The respondent was asked about acute diseases diagnosed by health professionals. Ten diseases included (i) Jaundice/ Hepatitis (ii) Tuberculosis (TB) (iii) Malaria (iv) Diarrhea/gastroenteritis (v) Typhoid (vi) Urinary Tract Infection (vii) Anaemia (viii) Chikungunya (ix) Dengue (x) Others. All the above questions on the scale of two e.g. 0 ‘No’ and 1 ‘Yes’. The scale of 0–10 was obtained by using *egen* command. The variable was recoded as 1 “Yes” if respondent has any of the diseases and 0 “No” otherwise. NCDs among older adults were assessed using eight chronic diseases diagnosed ever by any health professional. Eight chronic diseases included (i) Hypertension or high blood pressure, (ii) Diabetes or high blood sugar, (iii) Cancer or a malignant tumor, (iv) Chronic lung diseasesa such as asthma, chronic obstructive pulmonary disease/Chronic bronchitis or other chronic lung problems, (v) Chronic heart diseases such as Coronary heart disease (heart attack or Myocardial Infarction), congestive heart failure, or other chronic heart problems, (vi) Stroke, (vii) Arthritis or rheumatism, Osteoporosis or other bone/joint diseases, (viii) High cholesterol. All the above questions on a scale of two, e.g., 0 “No” and 1 “Yes”. The scale of 0–8 was obtained by using *egen* command. The variable was recoded as 1 “Yes” if the respondent has any of the diseases and 0 “No” otherwise.

Depressive symptoms among older adults were calculated using the Short Form Composite International Diagnostic Interview (CIDI-SF), with a score of 3 or more coded as 1 for “diagnosed with depression” and less than 3 coded as 0 for “not diagnosed with depression”. This scale estimates a probable psychiatric diagnosis of major depression and has been validated in field settings and is widely used in population-based health surveys [36]. Psychiatric disorder was coded as 1 if the respondent has been diagnosed with any neurological or psychiatric disorder (depression, Alzheimer’s disease, dementia, psychiatric problems such as unipolar/bipolar disorder, schizophrenia, neurological problems such as neuropathy, convulsions, migraine, Parkinson’s or others) and 0 otherwise.

#### Key explanatory variable

The main explanatory variable was experiencing natural and human-made disaster in the last five years. The question used for natural disasters was, “In the last five years, has your health been severely affected by disasters such as floods, landslides, extreme cold and hot weather, cyclones/typhoons, droughts, earthquakes, tsunamis, or any other natural calamities?” Question used for human-made disaster was “In the last five years, has your health been severely affected by human-made incidents such as riots, terrorism, building collapses, fires, traffic accidents or any other human-made incidents?” It was dichotomized into 0, representing not experiencing any natural or human-made disaster, and 1, representing yes. Any disaster was created using the questions and coding, either natural or human-made, as 1 or 0.

#### Others explanatory variables

*Individual factors*. Age was categorized into groups of 60–69 years, 70–79 years and 80+ years. Sex was categorized as male and female. Educational status was categorized as no education/primary, secondary and higher. Work status was recoded into never worked, ever worked but currently not, and currently working.

*Household-related factors*. The monthly per capita expenditure (MPCE) quintile was assessed using household consumption data. Sets of 11 and 29 questions on the expenditures on food and non-food items, respectively, were used to canvas the sample households. Food expenditure was collected based on a reference period of seven days, and non-food expenditure was collected based on reference periods of 30 days and 365 days. Food and non-food expenditures have been standardized to the 30-day reference period. The MPCE is computed and used as the summary measure of consumption [34]. The variable was then divided into three quintiles, i.e., poor, middle, and rich. Caste was recoded as Scheduled Caste (SC), Scheduled Tribe (ST), Other Backward Class (OBC), and others. The Scheduled Caste includes a group of population that is socially and economically segregated and deprived. The OBC is the group of people who were identified as “educationally, economically and socially backward”. The “other” caste category is identified as those having higher social status. Religion was coded as Hindu, Muslim, and Others.

*Community-related factors*. Mean education in PSUs was calculated as average educational level of respondents in each PSU. Mean wealth in PSUs was calculated as the average MPCE quintile of each PSU. Distance to primary healthcare was assessed using the community survey question. Immunization in PSUs included receiving influenza vaccine, pneumococcal vaccine, hepatitis B vaccine, typhoid vaccine, Diphtheria and Tetanus (dT) or any other vaccine. Place of residence was categorized as rural and urban. The regions were coded as North, Central, East, Northeast, West, and South.

### Statistical analysis

Descriptive statistics and cross-tabulations were presented in the study. The multivariate logistic regression only allows us to examine the associations at single level and does not account for the variation at another level of nested data. Besides, some locations may be more prone to disasters and there are regional and community-level differences in vulnerability to disasters. Therefore, considering the hierarchical structure of LASI data, we used multivariate multilevel logistic regression analysis to examine the association between experience of disaster and various health outcomes (poor SRH, difficulty in ADL, difficulty in IADL, CDs, NCDs, depressive symptoms and psychiatric disorders).

Initially, we conducted fixed-effects multilevel logistic regression analysis, considering individuals (level 1) nested within households (level 2) nested within community (level 3). However, we encountered convergence difficulties during this process. One possible explanation for the lack of convergence in the estimates from the three-level multilevel model is the smaller sample size of household-level data, which reduced statistical power and increased the uncertainty of the estimates. Analyzing the distribution of household members in the data, we observed that the majority of households consisted of only one or two individuals (as LASI collected data mainly from older members and their spouses), with a small proportion having three or more individuals. Such a distribution introduces significant variability or heterogeneity in the household-level data, making it more challenging to estimate the parameters and achieve a stable solution accurately. Therefore, we only performed a two-level (individual and community-levels) random intercept logistic regression model for the current analysis (using *melogit* command in Stata). We included all the individual (age, sex, education and work status), household (wealth quintiles, caste and religion) and community characteristics (mean education, mean wealth, distance to primary healthcare, and immunization in PSUs) in the model. In a random intercept multilevel logistic regression model, we include an effect for each area that impacts on all individuals in that area equally, regardless of their background characterictics.


$$\text{logit}\left(\mathrm{Pr}\left(y_{ij}=1\right)=\beta_{0j}+\beta_{1}x_{1ij}+\beta_{2}x_{2j}+\ldots .+\beta_{p}x_{pij}+u_{0j}\right.$$

In the above equation, *y*_*ij*_ is our dependent or response variable: the outcome for individual *i* living in PSU *j*, the chances of experiencing disaster. The respondents are numbered from *i* = 1, …, *N* and each lives in one PSU *j* = 1, …, *J*. There are *n*_*j*_ respondents in PSU *j* so N = ∑ ^J^_j = 1_ n_*j*_. Further, *x*_*pij*_ are the independent or explanatory variables, again measured on individual *i* in PSU *j*. The subscript *p* is used simply to distinguish between the different variables; for example *x*_1*ij*_ might be the individual’s age in years and *x*_2*ij*_ a dummy variable indicating the subject’s sex (0 = male, 1 = female). *x*_*pj*_ are also independent variables, but these are measured at the contextual or PSU level; that is, they take the same value for all individuals living in PSU *j*. These variables may be directly observed or measured at the PSU level; for example, *x*_3*j*_ may be the distance to primary healthcare in PSU *j*. Alternatively, the contextual variables may represent an aggregation of individual measures; *x*_4*j*_ may be the mean education of the respondents in PSU *j*.

*β*_*p*_ is the regression coefficient associated with *x*_*pij*_ or *x*_*pj*_. So *β*_1_ would indicate the average change in disaster associated with a 1-year increase in age and *β*_2_ would show the average effect of being female on experiencing disaster (relative to that for the baseline category, male, for which *x*_2*ij*_ = 0). *u*_0*j*_ is the estimated effect or residual for area *j*. This is the difference that we expect to see in experiencing disaster for an individual in PSU *j* compared to an individual in the average PSU, after taking into account those (individual, household and community) characteristics that have been included in the model. The 0 in the subscript denotes that this is a *random intercept* residual, a departure from the overall intercept *β*_0_ applying equally to everyone in PSU *j* regardless of individual’s characteristics. Such a model can offer a comprehensive framework for understanding the ways in which places or community level factors can affect the health outcomes associated with disaster among older adults, especially when the disaster experiences may vary across communities. While adjusting for such contextual effects, the intra-class correlation coefficients (ICC) are computed to quantify the amount of design effect present.

The models are diagnosed using the Log-likelihood Ratio test (LR test), Akaike’s Information Criterion (AIC) and Bayesian information criterion (BIC), and the results from the model diagnostics including ICC are presented in S1 Table. Stata version 15 has been used throughout the analysis [37]. The individual weights were used for computing the estimates to account for complex survey design and to make the estimates nationally representative.

#### Multivariate multilevel analysis fit statistics

ICC, LR test, AIC and BIC were calculated for the appropriateness of multilevel logistic regression and checking the presence of clustering. To choose the appropriate model for the current study, we first fit the null model to examine the between community variation and justify the use of multilevel analysis and then a model were built with dependent variable and individual level factors. Accordingly, the measures of community variation (obtained from random-intercept model) were estimated as the ICC and the value was significant in case of all the health outcomes considered in this study. We used ICC value greater than 5% to consider a variation of natural and human-made disasters across the clusters (S1 Table).

## Results

### Socio-demographic profile and state-wise prevalence of natural and/or human-made disaster in the study sample

Table 1 presents the socio-economic profile of older adults in India. About 11% of older adults were from age group 80 years and above. The mean age of the study population was 69.2 years. Nearly 74% of the older adults had no education or were educated till primary. Almost 35% of the older adults were currently not working whereas about 9% were retired. Mean years of education in the PSU was 3.4 years and mean wealth quintile of the PSUs was 1.5. Mean distance from to primary health care centre in PSUs were 2.3 km. Around 6.42% were immunized and 28.78% were urban residents.

**Table 1:** Socio-economic profile of the sample population.

Background characteristics	n (w %) or mean (SD)
**Individual factors**	
Age (in years)	
60–69	17627 (58.46)
70–79	8514 (30.22)
80+	3192 (11.32)
Sex	
Male	14120 (47.44)
Female	15213 (52.56)
Education	
No	16077 (56.69)
Primary	5322 (17.54)
Secondary	5554 (18.18)
Higher	2380 (7.6)
Work status	
Never	7932 (26.25)
Ever	11078 (37.88)
Currently	10323 (35.86)
**Household-related factors**	
MPCE quintile	
Poor	12168 (43.47)
Middle	5985 (20.94)
Rich	11180 (35.59)
Caste	
SC/ST	9242 (27.01)
OBC	11771 (46)
others	8320 (27.39)
Religion	
Hindu	22136 (82.43)
Muslim	3083 (11.24)
Others	4114 (6.33)
**Coomunity-related factors**	
Mean education in PSU (in years)	3.40 (4.61)
Mean wealth in PSU (1–5 quintiles)	1.50 (0.50)
Mean distance from primary healthcare (in kilometers)	2.31 (1.00)
Immunization in PSU	
No	26572 (93.58)
Yes	2761 (6.42)
Place of residence	
Rural	20052 (71.22)
Urban	9281 (28.78)
Region	
North	5317 (12)
Central	4262 (21.21)
East	5757 (23.93)
Northeast	3778 (2.87)
west	3666 (17.26)
South	6553 (22.94)
**Total**	29333 (100)

Notes: MPCE: Monthly per capita consumption expenditure; SC/ST: Scheduled caste/scheduled tribe; OBC: Other backward classes; PSU: Primary sampling unit

Table 2 presents the state-wise prevalence of natural and human-made disasters that affected the health of older individuals, LASI, wave-1. Overall, 3.58% of older adults reported that they have encountered any type of natural or human-made disaster whereas, 2.70% reported some type of natural disaster and 1.07% reported some type of human-made disaster in the past five years. The prevalence of natural disasters that affected the health of the older individuals was highest in Bihar (6.69%) followed by Madhya Pradesh (5.42%) and Uttar Pradesh (5.99%). Moreover, the prevalence of human-made disasters that affected the health of the older individuals was highest in Jammu and Kashmir (4.30%) followed by Madhya Pradesh (2.45%) and Bihar (1.89%).

**Table 2:** State-wise prevalence of natural, human-made and any disaster.

State name	Natural disaster	Man-made disaster	Any disaster	Total sample
	n (w %)	n (w %)	n (w %)	n
Andhra Pradesh	20 (2.21)	12 (1.01)	32 (3.23)	1,105
Arunachal Pradesh	4 (0.72)	5 (1.82)	9 (2.54)	318
Assam	11 (1.22)	9 (0.99)	20 (2.20)	816
Bihar	139 (6.69)	19 (0.89)	149 (7.19)	1,808
Chandigarh	2 (0.64)	2 (1.27)	4 (1.91)	394
Chhatisgarh	20 (2.27)	10 (1.26)	30 (3.53)	780
Dadra and Nagar Hav‥	1 (0.26)	7 (1.49)	8 (1.75)	451
Daman and Diu	7 (0.99)	1 (0.52)	8 (1.52)	434
Gujarat	9 (0.59)	14 (1.06)	21 (1.50)	991
Haryana	6 (0.58)	2 (0.17)	7 (0.65)	848
Himachal Pradesh	19 (2.9)	6 (1.08)	20 (3.04)	621
Jammu and Kashmir	34 (5.37)	28 (4.3)	48 (7.12)	731
Jharkhand	48 (4.27)	11 (0.99)	57 (5.14)	1,168
Karnataka	12 (0.72)	18 (0.76)	28 (1.36)	1,004
Kerala	12 (0.59)	8 (0.23)	20 (0.82)	1,209
Madhya Pradesh	71 (5.99)	37 (2.45)	99 (7.90)	1,313
Maharashtra	15 (0.84)	9 (0.58)	23 (1.36)	1,790
Manipur	10 (1.4)	6 (1.03)	16 (2.43)	606
Mizoram	15 (3.03)	4 (0.35)	16 (3.10)	531
Nagaland	13 (3)	0	13 (3.00)	608
Odisha	37 (3.22)	9 (0.9)	46 (4.13)	1,237
Puducherry	0	11 (1.3)	11 (1.30)	640
Punjab	3 (0.25)	10 (0.81)	13 (1.06)	1,004
Rajasthan	18 (1.32)	10 (0.94)	28 (2.26)	1,078
Sikkim	9 (1.86)	2 (0.5)	10 (2.11)	438
Tamil Nadu	69 (2.25)	8 (0.5)	77 (2.75)	1,534
Telangana	12 (1.2)	10 (1.28)	22 (2.47)	1,061
Tripura	3 (0.58)	5 (1.16)	8 (1.75)	461
Uttar Pradesh	115 (5.42)	39 (1.85)	143 (6.61)	2,169
Uttarakhand	6 (1.03)	8 (0.95)	14 (1.99)	641
West Bengal	10 (0.84)	9 (0.75)	17 (1.42)	1,544
Total	750 (2.74)	329 (1.08)	1017 (3.58)	29,333

### Natural and/or human-made disaster and associated health outcomes among older adults

Table 3 presents the prevalence of health outcomes of older individuals who experienced / did not experience natural and human-made disaster. Compared to those who did not experience any (natural or human-made) disaster, older adults who experienced any disaster had a higher prevalence of poor SRH (33.4% vs 23.31%), difficulty in ADL (33.94% vs 23.00%), difficulty in IADL (60.09% vs 47.70%), CDs (49.57% vs 25.86%), depressive symptoms (17.30% vs 8.06%) and psychiatric disorders (3.42% vs 2.78%).

**Table 3:** Prevalence of different health outcomes among those who experienced/ did not experience natural, human-made and any disaster.

Health outcomes	Natural disaster			Human-made disaster				Any disaster	
	No	Yes	Chi-square p-value	No	Yes	Chi-square p-value	No	Yes	Chi-square p-value
	n (w %)	n (w %)		n (w %)	n (w %)		n (w %)	n (w %)	
**Poor SRH**	6472 (23.37)	232 (34.39)	<0.001	6580 (23.53)	124 (36.80)	<0.001	6379 (23.31)	325 (33.54)	<0.001
**Difficulty in ADL**	5907 (23.29)	228 (34.18)	<0.001	6023 (23.47)	112 (35.00)	<0.001	5821 (23.00)	314 (33.94)	<0.001
**Difficulty in IADL**	12704 (47.77)	420 (61.56)	<0.001	12938 (48.00)	186 (58.47)	<0.001	12560 (47.70)	564 (60.09)	<0.001
**CDs**	6886 (25.95)	360 (53.69)	<0.001	7121 (26.55)	125 (41.36)	<0.001	6793 (25.86)	453 (49.57)	<0.001
**NCDs**	15296 (53.03)	421 (51.29)	0.156	15524 (52.99)	193 (52.82)	0.063	15145 (53.05)	572 (51.12)	0.083
**Depressive symptoms**	1921 (8.10)	104 (18.77)	<0.001	1980 (8.32)	45 (14.87)	<0.001	1888 (8.06)	137 (17.30)	<0.001
**Psychiatric disorders**	771 (2.78)	29 (3.54)	0.520	782 (2.79)	18 (3.92)	0.002	759 (2.78)	41 (3.42)	0.009

Notes: SRH: Self-rated health; ADL: Activities of daily living; IADL: Instrumental activities of daily living; CD: Communicable diseases; NCD: Non-communicable diseases

Table 4 presents the multivariate random intercept multilevel logistic regression estimates of health outcomes affected by natural and human-made disaster among older adults. The odds of poor SRH were significantly higher among older adults who experienced natural disaster [AOR: 1.51; CI: 1.26–1.81] or human-made disaster [AOR: 2.19; CI: 1.70–2.82] in comparison to older adults who did not experience natural or human-made disaster, respectively. The odds of difficulty in ADL and IADL were significantly higher among older adults who experienced natural disaster [AOR: 1.78; CI: 1.47–2.14 and AOR: 1.58; CI: 1.32–1.88] or human-made disaster [AOR: 2.18; CI: 1.68–2.84 and AOR: 1.79; CI: 1.39–2.31] in comparison to those who did not experience natural or human-made disaster. The odds of CDs and NCDs were significantly higher among older adults who suffered from any natural disaster [AOR: 2.37; CI: 2.00–2.82 and AOR: 1.44; CI: 1.22–1.70] or human-made disaster [AOR: 1.65; CI: 1.28–2.12 and AOR: 1.34; CI: 1.05–1.71] in comparison to those who did not experience natural or human-made disaster. The odds of depressive symptoms and psychiatric disorder were significantly higher among older adults who experienced natural disaster [AOR: 1.60; CI: 1.26–2.03 and AOR: 1.45; CI: 1.01–2.18] or human-made disaster [AOR: 1.89; CI: 1.33–2.67 and AOR: 2.07; CI: 1.24–3.47] in comparison to those who did not experience natural or human-made disaster, respectively.

**Table 4:** Multivariate multilevel logistic regression estimates of health outcomes by natural, human-made and any disaster.

	Natural disaster	Human-made disaster	Any disaster
Health outcome	AOR (95% CI)	AOR (95% CI)	AOR (95% CI)
Poor SRH	1.51***(1.26, 1.81)	2.19***(1.70, 2.82)	1.64***(1.40, 1.92)
Difficulty in ADL	1.78***(1.47, 2.14)	2.18***(1.68, 2.84)	1.89***(1.61, 2.21)
Difficulty in IADL	1.58***(1.32, 1.88)	1.79***(1.39, 2.31)	1.63***(1.40, 1.89)
CDs	2.37***(2.00, 2.82)	1.65***(1.28, 2.12)	2.12***(1.83, 2.46)
NCDs	1.44**(1.22, 1.70)	1.34*(1.05, 1.71)	1.38***(1.20, 1.60)
Depressive symptoms	1.60**(1.26, 2.03)	1.89***(1.33, 2.67)	1.67***(1.55, 2.05)
Psychiatric disorders	1.45*(1.01, 2.18)	2.07**(1.24, 3.47)	1.52***(1.33, 2.18)

Notes: *p<0.1; **p<0.05; ***p<0.01; SRH: Self-rated health; ADL: Activities of daily living; IADL: Instrumental activities of daily living; CD: Communicable diseases; NCD: Non-communicable diseases; All models are adjusted for various individual (Age, Sex, Education, Work-Status)-, household (MPCE quintile, Caste, Religion, Region)-, and community (Mean education in the PSU, Mean wealth in the PSU, Mean distance from primary health care, Place of residence)-level characteristics. The estimates are after excluding states/ union territories with equal to or less than 5 cases reporting the experience of natural or human made disaster, i.e., Arunachal Pradesh, Dadra and Nagar Haveli, Daman and Diu, Haryana, Mizoram, Nagaland, Puducherry, Punjab, Sikkim and Tripura.

Table 4 also presents the multivariate multilevel logistic regression estimates of health outcomes associated with any (natural or human-made) disaster among older adults. After accounting for various individual-, household-, and community variables, the estimates suggest that older adults who experienced any disaster were associated with poor SRH [AOR: 1.64; CI: 1.40, 1.92], difficulty in ADL and IADL [AOR: 1.89; CI: 1.61, 2.21 and AOR: 1.63; CI: 1.40, 1.89], CDs and NCDs [AOR: 2.12; CI: 1.83, 2.46 and AOR: 1.38; CI: 1.20, 1.60], depressive symptoms and psychiatric disorder [AOR: 1.67; CI: 1.55, 2.05 and AOR: 1.52; CI: 1.33, 2.18]. S2 Table presents the results from the multivariate multilevel logistic regression analyses of health outcomes by natural and/or human-made disasters and by individual-, household-, and community-level characteristics of older adults.

## Discussion

We assessed the prevalence of natural and human-made disasters and the association between such disasters and SRH, functional limitations, CDs and NCDs, depression symptoms and, psychiatric disorders among older adults in India. We found that about 4% of older Indians had experienced some natural or human-made disaster within the past five years. Importantly, the experience of these disasters was significantly linked to adverse physical, mental, and functional health outcomes among older Indians. Even after considering several individual, household, and community-related characteristics, we find that the risk of major depression among older Indians who had faced natural or human-made disasters was nearly double that of those who had not been exposed. These findings are consistent with previous studies [38–41], which also have highlighted the profound impact of disasters on mental health. The estimated prevalence of major depression among disaster-affected individuals in this study was nearly 18%. In other studies, the prevalence of major depression among survivors of the earthquake in Taiwan was 6.4% [42], while in Turkey, it was 11.0% [43], which is comparatively lower than that observed in our study. Bandla et al. (2019) reported that the depression prevalence in people living in flood-affected areas in Tamil Nadu, India, was 45%, which is relatively higher than in our study [44]. These differences may partially be attributed to the use of different diagnostic tools for major depression.

Research on the psychopathological consequences of disasters in older adults is gaining traction given the growing aging population exposed to such events. Disasters exert long-term effects, including lingering mental distress. Human-made disasters may affect individuals psychologically up to 6–14 years after the disaster, while coping with natural disasters can take up to 3 years after the event [45]. Thirty-seven years after the Tangshan earthquake in Japan, exposed persons were at greater risk of depression [46]. Severe earthquakes can lead to as deaths of kin and non-kin, triggering both acute and chronic mental distress [47]. Disaster related distress, however, varies across age. According to FCT, older adults may be particularly vulnerable to mental distress in the aftermath of disasters because of factors such as reduced capacity to mobilize assistance and a heightened sense of insecurity stemming from personal, social, and physical losses [48, 49]. Therefore, FCT underscores the importance of addressing social inequalities and improving SES to mitigate the adverse mental health effects of disasters among older adults. By reducing discrimination and improving SES, communities can better promote health and well-being by addressing both the individual and contextual factors that contribute to mental illness prevention [50].

In addition to psychological distress, we found that individuals who faced natural or human-made disasters were more likely to report functional deficits, as measured using ADLs and IADLs. Disasters increase the vulnerability of older adults to functional disabilities [51, 52]. This increased vulnerability to functional constraints among disaster survivors is consistent with FCT, which suggests that disasters worsen pre-existing social inequities and raise the risk of adverse health outcomes. For instance, and consistent with our findings, a cross-sectional study on the aftermath of the Bam earthquake in Iran shows that survivors report greater ADL limitations than non-exposed peers five years after the disaster [53]. Similarly, research conducted in China following the Wenchuan earthquake revealed decreased ADL capacity and muscle force among survivors than peers who remained unexposed [54]. Disasters often disrupt social interactions, such as separation from family and friends due to residential relocation, which can negatively impact functional health [55]. As expected, in our study, the survivors of natural or human-made disasters reported a greater likelihood of poor SRH than the non-exposed individuals. These findings align with previous research [56, 57] indicating a significant independent association between functional and mental health with poor SRH [58]. The potential mechanisms linking disasters and poor SRH in survivors, as evident in previous studies, include a various stressors, like post-disaster economic deprivation [59], breakdown of social networks [60], and reduced mental and physical capacities [61], underscoring the complex interplay between social conditions and health outcomes in disaster-affected populations.

Disaster survivors in our study also were at greater risk of CDs and NCDs. Previous literature corroborates our findings, indicating that individuals exposed to major disasters remain susceptible to developing comorbid conditions, such as hypertension, cancer, stroke, diabetes, and chronic lung disease [62]. A recent systematic review highlighted a greater prevalence of chronic conditions, including diabetes mellitus and hypertension, among individuals exposed to disasters such as storms, earthquakes, and major terrorist events like the World Trade Center Tower (WTCT) attack [63]. Exposure to polluted air and toxic chemicals during the WTCT attack has been linked to neurological conditions, certain types of cancer, and respiratory conditions such as asthma, chronic obstructive lung disease, pulmonary fibrosis, and pneumonia [64]. Disaster survivors often suffer from a multitute of exacerbated CDs and NCDs, likely attributable to adverse post-disaster conditions, like compromised sanitation, inadequate nutrition, and contaminated drinking water, temperature extremes, rise in infections, and disrupted health services [65]. For instance, a study conducted in Indonesia in 1992–93 revealed a significant increase in the risk of diarrheal illnesses, including paratyphoid fever, following the flooding [66]. Similarly, the South Asia earthquake in Pakistan in 2005 triggered multiple contiguous clusters of measles [67], underscoring the increased susceptibility to infectious diseases among those affected by disasters. These findings highlight the need for comprehensive public interventions targeted at both acute health needs and long-term health outcomes in disaster-prone communities.

### Policy implications

Both natural and human-made calamities exert disastrous health impacts on older adults. Considering FCT, which underscores the role of social inequities in shaping health outcomes, one way to improve both disaster preparedness and post-disaster management is to actively engage older adults as stakeholders in disaster preparedness and management [68]. Instead of victimizing older adults, programs and policies should prioritize promoting healthy aging, and invest in developing age-friendly communities. By fostering environments that support healthy aging, such initiatives not only build individual resilience but also empower older adults to confront disasters with greater self-confidence and facilitate post-disaster coping [68]. Furthermore, strengthening health care systems is crucial in mitigating the impact of disasters on older adults. Stronger health care systems would increase the capacity to manage the unexpected influx of older patients during disasters, ensuring timely and effective care delivery.

## Conclusions

This study underscores the increased vulnerability of older survivors of natural or human-made disasters within the past five years to physical and mental health challenges. Specifically, compared to their non-exposed counterparts, older survivors of natural or human-made disasters endure an inflated risk of SRH, functional difficulties, chronic CDs and NCDs, depressive symptoms, and psychiatric disorders. These findings call for a comprehensive, age-sensitive approach to interventions aimed at restoring the physical, mental and psychosocial well-being of survivors in the aftermath of a disaster. With age, people spend more time in their immediate neighborhoods and become increasingly dependent on local resources and services. As such, on a policy and intervention front, there is a need for more local and community-based disaster preparedness, improved warning systems and coordinated disaster responses, including rescue, relief, and accessible shelter options, especially in areas with substantial older adult populations.

### Strengths and limitations

As far as we know, this is the first study to assess the relevance of natural versus human-made disasters for such a wide variety of physical, functional, and mental health outcomes among older Indians, and we did so by employing a sizeable nationally representative sample of older adults. Moreover, we used standardized and validated measures, adapted from the US based Health and Retirement Study, for assessing outcomes of physical and psychological health. That said, our study has several limitations. First, our key explanatory variable, the experience of disasters, is assessed retrospectively, therefore prone to recall bias and misreporting. Second, the study’s cross-sectional nature prevents us from rendering any causal or temporal inferences about disaster exposure, and its health implications. Future studies using upcoming waves of LASI may be able to offer a more clarifying image of the relationship between disaster experience and health. Because some health concerns may not emerge immediately after exposure but instead take several years to surface [64], a health trajectories approach may offer greater opportunities for early intervention to maintain physical and mental potency among older disaster survivors. Finally, the inherent bias in the self-reporting of some of the outcome variables such as CDs and NCDs and SRH may have influenced the current results.

## Supporting information

S1 Table Model diagnostics of the multivariate multilevel logistic regression analyses of health outcomes by natural and/or human-made disasters among older adults. (DOCX)

S2 Table Multivariate multilevel logistic regression analyses of health outcomes by natural or human-made disasters and by background characteristics of older adults. (DOCX)
