# Supplementary material for: Natural and human-made disaster and associated health outcomes among community-dwelling older adults in India: Findings from LASI, 2017–18
Source: PLoS One. 2024 Jul 18;19(7):e0307371. doi: 10.1371/journal.pone.0307371 (PMC11257249; doi:10.1371/journal.pone.0307371)
Supplement: S1 Table — (DOCX) [file pone.0307371.s001.docx]

| **S1 Table: Model diagnostics of the multivariate multilevel logistic regression analyses of health outcomes by natural and/or human-made disasters among older adults** | | | |
| --- | --- | --- | --- |
|  | **Natural disasters** | **Human-made disasters** | **Natural or human-made disasters** |
| **Health outcome** |  |  |  |
| **Poor SRH** |  |  |  |
| Community level variance | 0.572(0.037) | 0.572(0.037) | 0.57(0.037) |
| Log-likelihood | -14587.136 | -14579.306 | -14577.658 |
| LR test χ2 | 810.24 | 809.71 | 807.98 |
| P > χ2 | 0 | 0 | 0 |
| ICC | 0.15 | 0.15 | 0.15 |
| AIC | 29232.27 | 29216.61 | 29213.32 |
| BIC | 29472.58 | 29456.92 | 29453.62 |
| **Difficulty in ADL** |  |  |  |
| Community level variance | 0.580(0.038) | 0.58(0.038) | 0.58(0.038) |
| Log-likelihood | -13602.622 | -13604.45 | -13590.797 |
| LR test χ2 | 774.87 | 769.74 | 770.68 |
| P > χ2 | 0 | 0 | 0 |
| ICC | 0.15 | 0.15 | 0.15 |
| AIC | 27263.24 | 27266.9 | 27239.59 |
| BIC | 27503.55 | 27507.21 | 27479.9 |
| **Difficulty in IADL** |  |  |  |
| Community level variance | 0.615(0.035) | 0.614(0.034) | 0.615(0.035) |
| Log-likelihood | -17661.659 | -17664.763 | -17654.542 |
| LR test χ2 | 1207.13 | 1204.90 | 1204.78 |
| P > χ2 | 0 | 0 | 0 |
| ICC | 0.16 | 0.16 | 0.18 |
| AIC | 35381.32 | 35387.53 | 35367.08 |
| BIC | 35621.63 | 35627.83 | 35607.39 |
| **CD** |  |  |  |
| Community level variance | 0.505(0.038) | 0.516(0.035) | 0.506(0.034) |
| Log-likelihood | -14945.896 | -14986.803 | -14945.254 |
| LR test χ2 | 686.39 | 714.87 | 686.14 |
| P > χ2 | 0 | 0 | 0 |
| ICC | 0.13 | 0.14 | 0.13 |
| AIC | 29949.79 | 30031.61 | 29948.51 |
| BIC | 30190.1 | 30271.91 | 30188.82 |
| **NCD** |  |  |  |
| Community level variance | 0.253(0.020) | 0.251(0.020) | 0.252(0.020) |
| Log-likelihood | -18442.157 | -18448.915 | -18441.491 |
| LR test χ2 | 355.05 | 352.23 | 353.39 |
| P > χ2 | 0 | 0 | 0 |
| ICC | 0.07 | 0.07 | 0.07 |
| AIC | 36942.31 | 36955.83 | 36940.98 |
| BIC | 37182.62 | 37196.14 | 37181.29 |
| **Depression symptoms** |  |  |  |
| Community level variance | 0.749(0.070) | 0.760(0.071) | 0.75(0.070) |
| Log-likelihood | -6953.519 | -6954.6188 | -6949.8303 |
| LR test χ2 | 320.05 | 329.37 | 319.12 |
| P > χ2 | 0 | 0 | 0 |
| ICC | 0.19 | 0.19 | 0.19 |
| AIC | 13965.04 | 13967.24 | 13957.66 |
| BIC | 14205.35 | 14207.55 | 14197.97 |
| **Psychiatric disorders** |  |  |  |
| Community level variance | 0.96(0.122) | 0.960(0.122) | 0.96(0.012) |
| Log-likelihood | -3464.4173 | -3462.571 | -3463.28 |
| LR test χ2 | 157.62 | 157.13 | 157.36 |
| P > χ2 | 0 | 0 | 0 |
| ICC | 0.23 | 0.23 | 0.23 |
| AIC | 6986.835 | 6983.142 | 6984.57 |
| BIC | 7227.142 | 7223.45 | 7224.88 |
| Notes: *p<0.1; **p<0.05; ***p<0.01; AOR: Adjusted Odds Ratio; SRH: Self-rated health; ADL: Activities of daily living; IADL: Instrumental activities of daily living; CD: Communicable diseases; NCD: Non-communicable diseases; AIC: Akaike information criterion; BIC: Bayesian information criterion; ICC: Intra class correlation; All models are adjusted for various individual-, household-, and community-level characteristics. | | | |
