# Supplementary material for: Natural and human-made disaster and associated health outcomes among community-dwelling older adults in India: Findings from LASI, 2017–18
Source: PLoS One. 2024 Jul 18;19(7):e0307371. doi: 10.1371/journal.pone.0307371 (PMC11257249; doi:10.1371/journal.pone.0307371)
Supplement: S2 Table — (DOCX) [file pone.0307371.s002.docx]

| **S2 Table: Multivariate multilevel logistic regression analyses of health outcomes by natural or human-made disasters and by background characteristics of older adults** | | | | | | | |
| --- | --- | --- | --- | --- | --- | --- | --- |
| **Background characteristics** | **Poor SRH** | **Difficulty in ADL** | **Difficulty in IADL** | **CD** | **NCD** | **Depression symptoms** | **Psychiatric disorders** |
|  | **AOR (95% CI)** | **AOR (95% CI)** | **AOR (95% CI)** | **AOR (95% CI)** | **AOR (95% CI)** | **AOR (95% CI)** | **AOR (95% CI)** |
| **Natural or human-made disaster** | |  |  |  |  |  |  |
| No | Ref. | Ref. | Ref. | Ref. | Ref. | Ref. | Ref. |
| Yes | 1.64***(1.41 -1.92) | 1.89***(1.61 -2.21) | 1.63***(1.40 -1.89) | 2.12***(1.83 -2.46) | 1.38***(1.20 -1.60) | 1.67***(1.55 -2.05) | 1.52***(1.33 -2.18) |
| **Age (in years)** |  |  |  |  |  |  |  |
| 60-69 | Ref. | Ref. | Ref. | Ref. | Ref. | Ref. | Ref. |
| 70-79 | 1.41***(1.32 -1.5) | 1.72***(1.61 -1.84) | 1.68***(1.59 -1.77) | 0.98 (0.92 -1.05) | 1.23***(1.16 -1.3) | 1.01 (0.9 -1.12) | 1.12 (0.95 -1.32) |
| 80+ | 1.91***(1.75 -2.08) | 3.1***(2.84 -3.38) | 3.02***(2.77 -3.29) | 0.95 (0.87 -1.05) | 1.08*(1 -1.17) | 1.03 (0.89 -1.21) | 1.69***(1.37 -2.07) |
| **Sex** |  |  |  |  |  |  |  |
| Male | Ref. | Ref. | Ref. | Ref. | Ref. | Ref. | Ref. |
| Female | 1.14***(1.07 -1.22) | 1.24***(1.16 -1.33) | 1.8***(1.69 -1.91) | 1.21***(1.13 -1.3) | 1.12***(1.06 -1.19) | 1.38***(1.24 -1.54) | 0.9 (0.76 -1.07) |
| **Education** |  |  |  |  |  |  |  |
| No/Primary | Ref. | Ref. | Ref. | Ref. | Ref. | Ref. | Ref. |
| Secondary | 0.89***(0.82 -0.96) | 0.82***(0.75 -0.89) | 0.55***(0.51 -0.59) | 0.84***(0.78 -0.92) | 1.26***(1.17 -1.35) | 0.9 (0.79 -1.03) | 0.99 (0.82 -1.21) |
| Higher | 0.58***(0.51 -0.66) | 0.63***(0.55 -0.72) | 0.38***(0.34 -0.42) | 0.69***(0.61 -0.78) | 1.43***(1.29 -1.59) | 0.76**(0.61 -0.94) | 0.92 (0.69 -1.22) |
| **Work status** |  |  |  |  |  |  |  |
| Never worked | Ref. | Ref. | Ref. | Ref. | Ref. | Ref. | Ref. |
| Ever worked but currently not | 1.15***(1.07 -1.25) | 1.09**(1.01 -1.18) | 1.3***(1.22 -1.4) | 1.16***(1.08 -1.26) | 0.97 (0.9 -1.04) | 1.3***(1.15 -1.48) | 0.99 (0.82 -1.21) |
| Currently working | 0.76***(0.7 -0.83) | 0.61***(0.55 -0.67) | 0.8***(0.74 -0.86) | 1.24***(1.14 -1.35) | 0.61***(0.57 -0.65) | 1.26***(1.1 -1.45) | 0.72***(0.57 -0.9) |
| **MPCE quintile** |  |  |  |  |  |  |  |
| Poor | Ref. | Ref. | Ref. | Ref. | Ref. | Ref. | Ref. |
| Middle | 0.88***(0.81 -0.95) | 1.01 (0.93 -1.1) | 0.92**(0.86 -0.98) | 1.03 (0.95 -1.11) | 1.22***(1.14 -1.3) | 0.88*(0.77 -1) | 1.18*(0.97 -1.44) |
| Rich | 1.02 (0.96 -1.09) | 1.04 (0.97 -1.11) | 0.99 (0.93 -1.04) | 1.19***(1.12 -1.27) | 1.55***(1.46 -1.64) | 1.17***(1.05 -1.3) | 1.26***(1.07 -1.49) |
| **Caste** |  |  |  |  |  |  |  |
| SC | Ref. | Ref. | Ref. | Ref. | Ref. | Ref. | Ref. |
| ST | 0.67***(0.6 -0.76) | 0.76***(0.67 -0.85) | 0.83***(0.75 -0.91) | 1.14**(1.03 -1.27) | 0.56***(0.51 -0.62) | 0.47***(0.38 -0.58) | 0.9 (0.66 -1.22) |
| OBC | 0.87***(0.8 -0.95) | 0.93*(0.85 -1.01) | 0.92**(0.85 -0.99) | 1.12***(1.03 -1.21) | 1.05 (0.97 -1.12) | 0.99 (0.87 -1.12) | 0.88 (0.71 -1.08) |
| Others | 0.9**(0.82 -0.98) | 1.04 (0.95 -1.14) | 1 (0.93 -1.09) | 0.9**(0.82 -0.98) | 1.15***(1.06 -1.24) | 0.9 (0.78 -1.04) | 0.92 (0.73 -1.16) |
| **Religion** |  |  |  |  |  |  |  |
| Hindu | Ref. | Ref. | Ref. | Ref. | Ref. | Ref. | Ref. |
| Muslim | 1.21***(1.1 -1.32) | 1.05 (0.95 -1.16) | 1.18***(1.09 -1.28) | 0.88**(0.8 -0.97) | 1.24***(1.14 -1.35) | 1.18**(1.02 -1.36) | 1.24*(0.99 -1.54) |
| Others | 1.02 (0.93 -1.13) | 0.92 (0.83 -1.02) | 0.85***(0.78 -0.93) | 0.8***(0.72 -0.89) | 1.19***(1.1 -1.3) | 1.09 (0.91 -1.29) | 0.96 (0.75 -1.24) |
| **Community education** |  |  |  |  |  |  |  |
| Illiterate | Ref. | Ref. | Ref. | Ref. | Ref. | Ref. | Ref. |
| Literate | 0.86***(0.77  0.96) | 0.87**(0.78  0.97) | 0.05 (1.16  0) | 1.26***(1.14  1.39) | 0.86***(0.79  0.93) | 0.08 (1.2  0) | 1.07 (0.86  1.34) |
| **Community wealth Index^¥^** |  |  |  |  |  |  |  |
| Poor | Ref. | Ref. | Ref. | Ref. | Ref. | Ref. | Ref. |
| Rich | 1.12**(1.01  1.24) | 1.07 (0.96  1.18) | 0.05 (1.21  0) | 1.12**(1.02  1.24) | 0.9***(0.84  0.97) | 0.08 (1.18  0) | 0.79**(0.64  0.97) |
| **Immunization in PSU** |  |  |  |  |  |  |  |
| No | Ref. | Ref. | Ref. | Ref. | Ref. | Ref. | Ref. |
| Yes | 0.79***(0.72  0.87) | 0.77***(0.7  0.85) | 0.04 (0.84  0) | 0.8***(0.73  0.88) | 0.73***(0.68  0.78) | 0.05 (0.87  0) | 0.64***(0.53  0.77) |
| **Distance from primary health care** |  |  |  |  |  |  |  |
| In village /CEB (>1 km) | Ref. | Ref. | Ref. | Ref. | Ref. | Ref. | Ref. |
| 1-5 km | 1.01 (0.9  1.14) | 1.05 (0.93  1.19) | 0.06 (1.24  0) | 1.03 (0.92  1.16) | 0.96 (0.88  1.05) | 0.1 (1.32  0) | 1.43***(1.13  1.82) |
| 6-10 km | 0.9 (0.78  1.04) | 1.03 (0.89  1.2) | 0.08 (1.23  0) | 1.04 (0.9  1.2) | 0.86***(0.77  0.95) | 0.1 (1.16  0) | 0.97 (0.71  1.32) |
| 10+ km | 0.89 (0.77  1.04) | 1.04 (0.89  1.21) | 0.08 (1.28  0) | 0.94 (0.81  1.09) | 0.9*(0.81  1.011 | 0.12 (1.31  0) | 1.26 (0.92  1.72) |
| **Place of residence** |  |  |  |  |  |  |  |
| Urban | Ref. | Ref. | Ref. | Ref. | Ref. | Ref. | Ref. |
| Rural | 1.01 (0.94 -1.08) | 1.06*(0.99 -1.14) | 1.37***(1.29 -1.45) | 1.29***(1.21 -1.38) | 0.61***(0.58 -0.65) | 1.29***(1.15 -1.44) | 0.8***(0.68 -0.94) |
| **Region** |  |  |  |  |  |  |  |
| North | Ref. | Ref. | Ref. | Ref. | Ref. | Ref. | Ref. |
| Central | 0.98 (0.89 -1.09) | 1.35***(1.21 -1.52) | 0.95 (0.87 -1.04) | 1.32***(1.21 -1.44) | 0.54***(0.49 -0.59) | 2***(1.72 -2.32) | 1.1 (0.82 -1.47) |
| East | 1.14***(1.04 -1.25) | 2.03***(1.83 -2.24) | 1.25***(1.15 -1.36) | 0.7***(0.64 -0.76) | 0.84***(0.78 -0.92) | 1.22**(1.05 -1.42) | 1.39**(1.08 -1.79) |
| Northeast | 0.69***(0.61 -0.78) | 1.1 (0.97 -1.25) | 0.86***(0.77 -0.95) | 0.3***(0.26 -0.34) | 0.75***(0.68 -0.83) | 0.53***(0.42 -0.67) | 0.89 (0.64 -1.25) |
| West | 0.68***(0.61 -0.77) | 2.87***(2.57 -3.2) | 1.13**(1.02 -1.24) | 0.62***(0.56 -0.69) | 1.14***(1.04 -1.25) | 1.08 (0.91 -1.3) | 1.1 (0.82 -1.49) |
| South | 1.93***(1.77 -2.12) | 1.51***(1.36 -1.68) | 1.64***(1.51 -1.78) | 0.29***(0.27 -0.32) | 1.48***(1.36 -1.61) | 0.85**(0.72 -0.99) | 2.38***(1.88 -3) |
| Pseudo R2 | 0.05 | 0.07 | 0.09 | 0.06 | 0.08 | 0.04 | 0.03 |
| Notes: *p<0.1; **p<0.05; ***p<0.01; AOR: Adjusted Odds Ratio; SRH: Self-Rated Health; ADL: Activities of daily living; IADL: Instrumental activities of daily living; MPCE: Monthly per capita consumption expenditure; SC: Scheduled caste; ST: Scheduled tribe; OBC: Other backward classes; ^¥^ Recoded as poor (poorer and poorest) and otherwise rich; PSU: Primary sampling unit; CEB: Census enumeration block | | | | | | | |
